# A case of metastatic renal cell carcinoma successfully treated with deferred cytoreductive nephrectomy following lenvatinib plus pembrolizumab combination therapy

**DOI:** 10.1002/iju5.12584

**Published:** 2023-03-19

**Authors:** Hiroaki Sato, Tomokazu Sazuka, Ayumi Fujimoto, Sakurako Kagitani, Takayuki Arai, Yusuke Goto, Yusuke Imamura, Shinichi Sakamoto, Jun‐Ichiro Ikeda, Tomohiko Ichikawa

**Affiliations:** ^1^ Department of Urology Chiba University Graduate School of Medicine Chiba Japan; ^2^ Department of Diagnostic Pathology Chiba University Graduate School of Medicine Chiba Japan; ^3^ Department of Molecular Pathology Chiba University Graduate School of Medicine Chiba Japan

**Keywords:** deferred cytoreductive nephrectomy, immuno‐oncology therapy, metastatic renal cell carcinoma, tyrosine kinase inhibitor

## Abstract

**Introduction:**

Combination therapy using immuno‐oncology drugs with tyrosine kinase inhibitors is increasingly important in the therapeutic strategy for metastatic renal cell carcinomas. Here, we report a case of metastatic renal cell carcinoma that was successfully treated with deferred cytoreductive nephrectomy following lenvatinib plus pembrolizumab combination therapy.

**Case presentation:**

A 49‐year‐old man was referred to our hospital with a diagnosis of advanced right kidney cancer with multiple lung metastases (cT3aN0M1). The size of the primary tumor was so huge that it exceeded 20 cm in diameter, pushing the liver and intestines to the left. After administration of lenvatinib and pembrolizumab combination as first‐line treatment, all the metastatic lung lesions disappeared, and the primary lesion shrank significantly. Robot‐assisted radical nephrectomy was successfully performed, resulting in complete surgical remission.

**Conclusion:**

Deferred cytoreductive nephrectomy following a lenvatinib plus pembrolizumab combination is a useful therapeutic strategy for achieving complete remission of metastatic renal cell carcinomas.

**Keynote message**

Achieving complete remission of metastatic renal cell carcinoma is challenging. In the era of immuno‐oncology drugs, deferred cytoreductive nephrectomy following combined immunotherapy with a tyrosine kinase inhibitor may be a useful therapeutic strategy in the management of metastatic renal cell carcinomas.

**Abbreviations & Acronyms**
CN: cytoreductive nephrectomy
CR: complete remission/complete response
CT: computerized tomography
DCN: deferred CN
IFN: interferon
IO: immuno‐oncology
mRCC: metastatic RCC
RCC: renal cell carcinoma
TKI: tyrosine kinase inhibitor

## Introduction

In association with the superiority of IO‐IO and IO‐TKI combination therapies seen in clinical trials,^1^, ^2^, ^3^, ^4^, ^5^ IO therapy has led to a paradigm shift in the treatment of mRCCs. Among them, the results of the lenvatinib plus pembrolizumab combination^5^ revealed not only improved survival but also remarkable tumor objective response rate. While the optimal sequence of CN and a systemic IO‐based combination is still unclear, for certain patients in the DCN setting, tumor shrinkage can reduce the degree of surgical invasiveness required, and contribute to surgical CR. Here, we present a case of primary mRCC successfully treated with DCN following lenvatinib plus pembrolizumab combination therapy.

## Case presentation

**Fig. 1:**
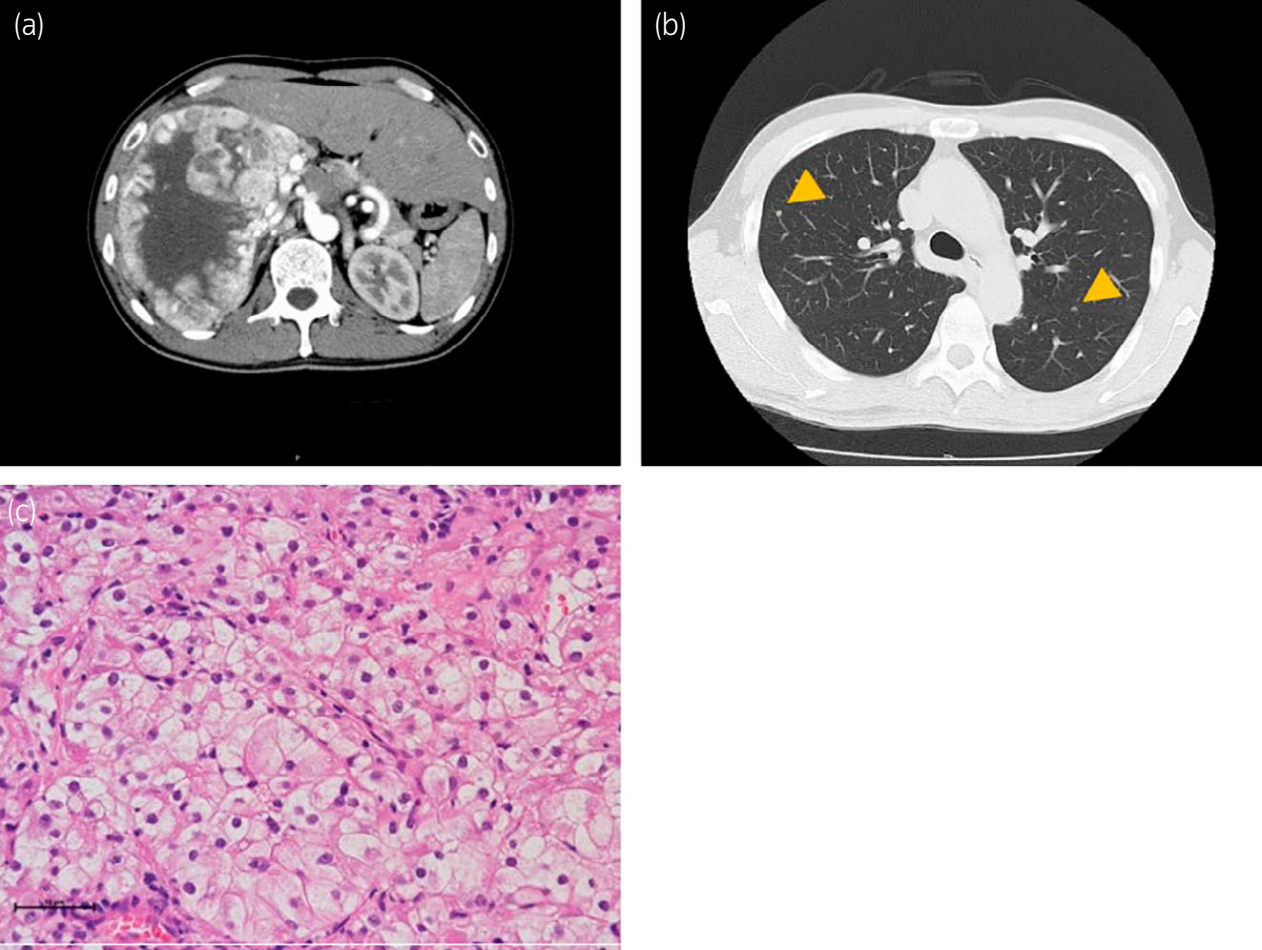
Clinical and histopathological findings at initial diagnosis. (a) Abdominal enhanced CT revealed huge (20 cm in diameter) right renal tumor with renal sinus and peri‐nephric fat involvement surrounded by abnormal neovascularization. (b) Chest CT revealed multiple lung nodules which were diagnosed as metastatic tumors (pointed by yellow arrows). (c) Pretreatment histological section of biopsy specimen demonstrated the pathological diagnosis of clear cell RCC. Compact nests of tumor cells with clear or pale cytoplasm are separated by delicate sinusoidal vascular networks.

**Fig. 2:**
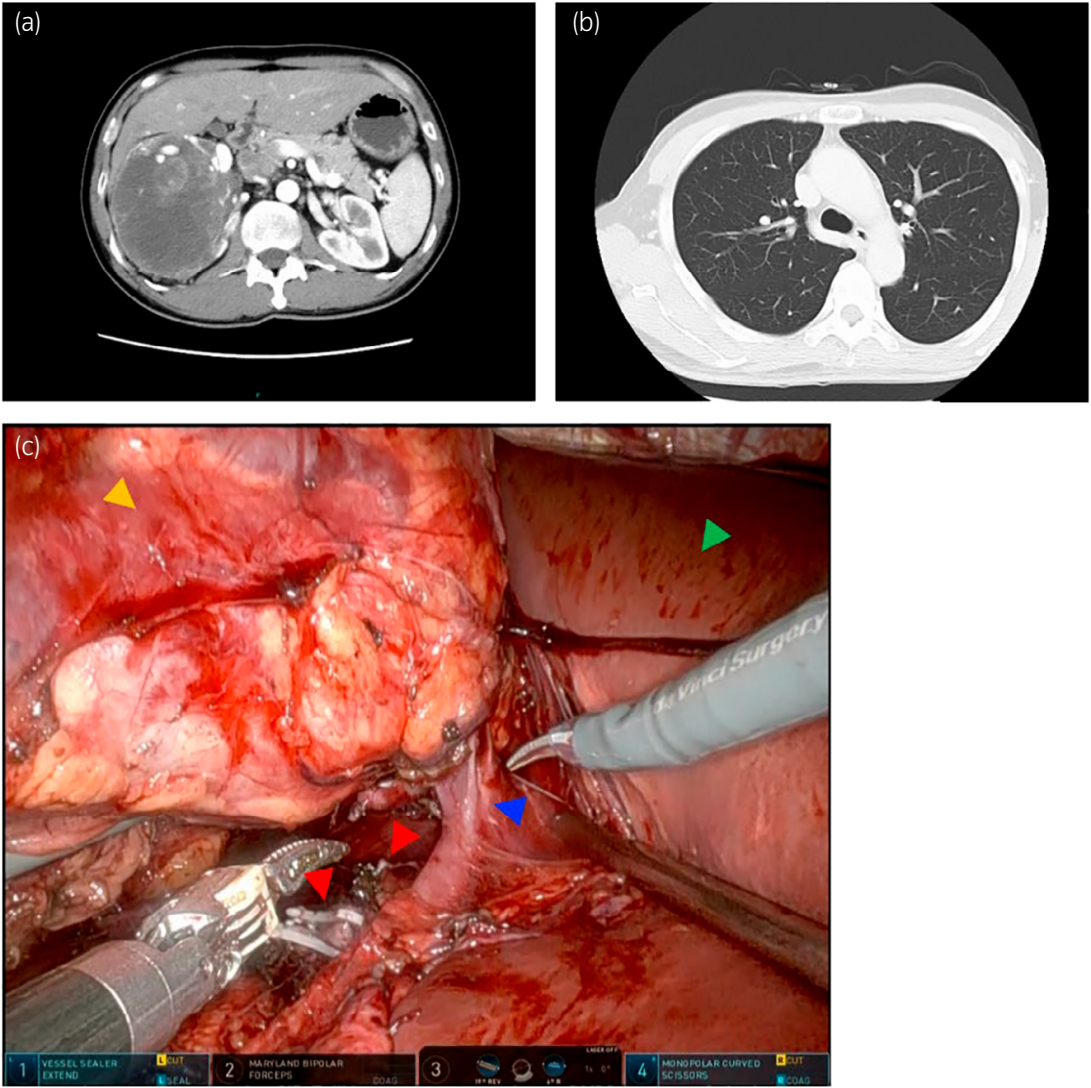
Clinical findings during the systematic treatment. (a) Abdominal enhanced CT performed in 19th week after the initiation of lenvatinib plus pembrolizumab combo. Primary tumor shrunk remarkably into 13 cm in diameter. (b) Disappearance of lung metastases was confirmed by chest CT. (c) Representative image from the surgical video. Primary tumor (yellow arrow), ligated and dismembered renal arteries (red arrows), renal vein (blue arrow), and liver (green arrow) were demonstrated during the robot‐assisted laparoscopic procedure.

**Fig. 3:**
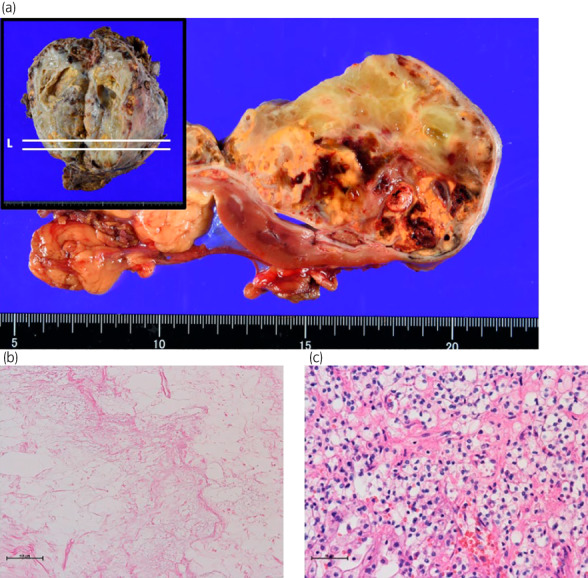
Histopathological findings of the posttreatment surgical specimen. (a) Gross findings of the specimen. White lines in the upper left panel demonstrated the split surface. Tumor infiltration into both renal sinus and peri‐nephric fat was observed. Marked myxoid degeneration was seen in the center of tumor whereas yellow solid nodules were present in the tumor periphery. (b) Coagulative necrosis in the center area of the specimen. (c) Posttreatment histological section of surgical specimen. The histological appearance resembles pretreatment biopsy (Fig. 1c).

**Fig. 4:**
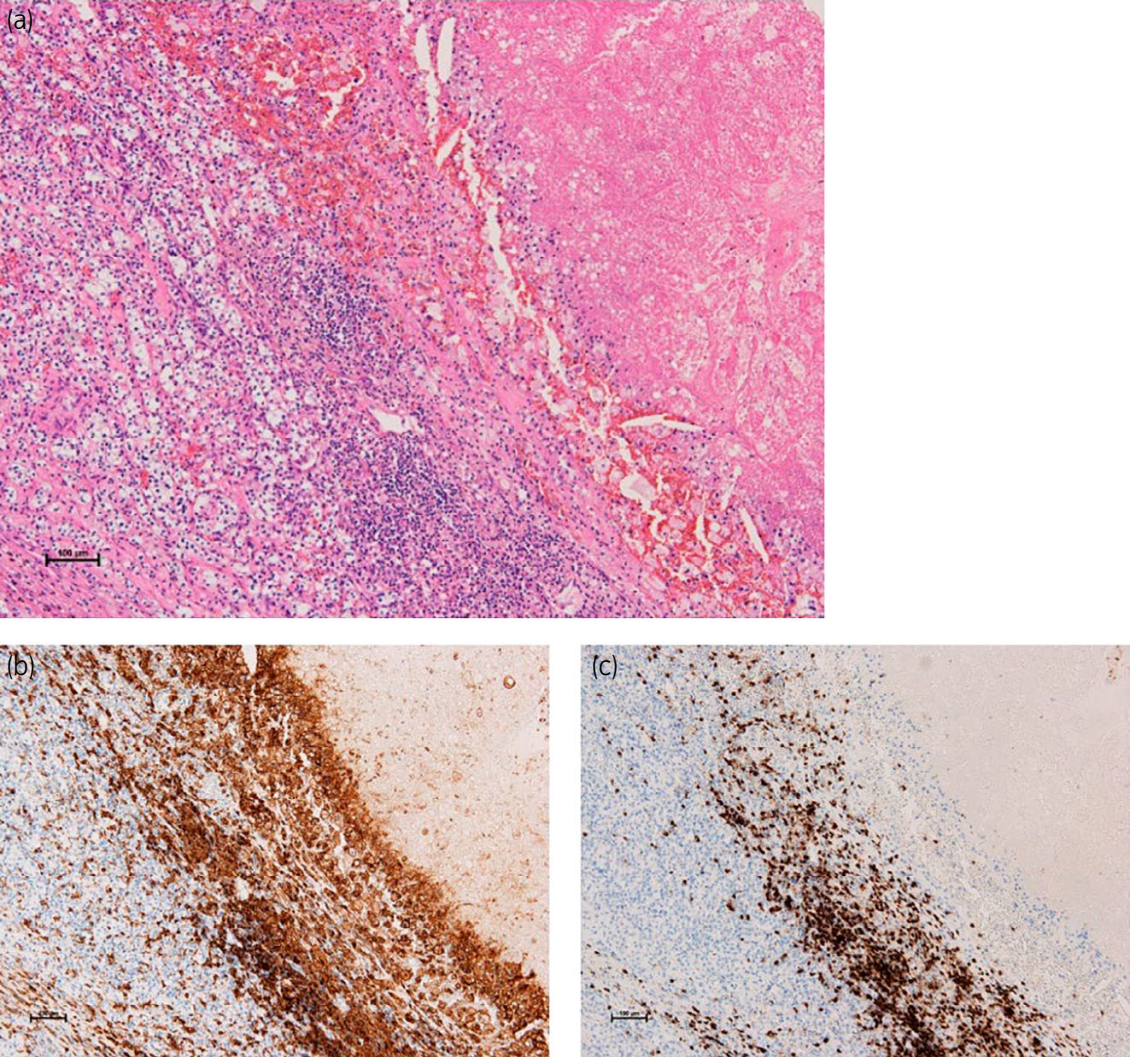
Immunohistopathological findings of the posttreatment surgical specimen. (a) Hematoxylin‐Eosin staining section representing viable tumor and necrosis. Coagulative necrosis and dense lymphocyte infiltration (right side) are juxtaposed to viable tumors (left side). (b, c) Immunohistochemical staining for CD4 (b) and CD8 (c). Infiltrating lymphocytes were predominantly CD4^+^ T‐cells.

A 49‐year‐old man was referred to our hospital with a diagnosis of advanced right kidney cancer. He had visited the former doctor because of asymptomatic gross hematuria. Urine cytology was negative. Enhanced CT revealed a large right kidney tumor exceeding 20 cm in diameter, which was surrounded by abnormal neovascularization, and was pushing the liver and intestines to the left (Fig. 1a). On chest CT, multiple lung nodules were observed (Fig. 1b), which the diagnostic radiology team at our hospital diagnosed as multiple metastases. Therefore, a clinical diagnosis of cT3aN0M1 mRCC was made. A percutaneous needle biopsy provided us the pathological diagnosis of clear cell RCC (Fig. 1c). Among six International Metastatic RCC Database Consortium (IMDC) risk factors, time from diagnosis to treatment was applicable while other five factors were not; Karnofsky performance status was 90% and hemoglobin, neutrophils, platelets, and corrected calcium level were within the normal range. We considered systemic therapy as suitable for this patient, because immediate CN for this huge primary tumor and total metastasectomy for multiple lung tumors seemed too invasive. In addition, since IO‐TKI combination was thought to be better suited for achieving remarkable tumor reduction than IO‐IO, lenvatinib plus pembrolizumab combination therapy was administered as the first line of treatment. Starting dose of lenvatinib and pembrolizumab was 14 and 400 mg, respectively. During systemic therapy, the starting dose was maintained and no severe adverse event was observed, although the patient had grade 2 hypertension, grade 2 hand–foot syndrome, grade 1 diarrhea and grade 1 elevated transaminase levels. Nine weeks after administration of the combination therapy, the lung metastases disappeared, and the primary tumor and surrounding neovascularization shrank remarkably as revealed by the CT scan. An additional 10 weeks of treatment maintained the complete remission of the lung metastases and resulted in further shrinkage of the primary tumor to 13 cm in diameter (Fig. 2a,b), leading to a decision to perform DCN by a robot‐assisted laparoscopic procedure. Twenty‐three weeks after the treatment initiation (final administration of pembrolizumab in 19th week, and cessation of lenvatinib on pre‐operative day 8), robot‐assisted radical nephrectomy was successfully performed (Fig. 2c). The operative time was 219 min, and the console time was 146 min. The estimated blood loss was 330 mL without blood transfusion. No major surgical complication was observed. Histopathological analysis (Fig. 3a–c) revealed ypT3a grade 2 clear cell RCC without sarcomatoid feature, and the surgical margin was negative. More than half of the specimen demonstrated coagulative necrosis, and viable cancer cells were observed in approximately 30% of the specimen. Dense lymphocyte infiltration was observed in juxtaposed necrosis and viable tumor tissue, suggesting an immune response activated by IO therapy (Fig. 4a). Immunohistochemistry confirmed CD4 and CD8 staining of these infiltrated lymphocytes (Fig. 4b,c). Surgical CR was achieved in this patient, and he is now in a treatment‐free state.

## Discussion

Combination therapies using immune checkpoint inhibitors such as IO‐IO and IO‐TKI have improved survival in mRCC^1^, ^2^, ^3^, ^4^, ^5^ and have been recognized as the standard first line of care for patients with mRCC.^6^, ^7^ As no definite biomarker has been established for the stratification of selecting which IO‐based combination should be prescribed, urologists and clinical oncologists must consider each patient's background, considering factors such as primary tumor size, location and burden of metastatic tumors, performance status, symptoms, age, and IMDC risk. In addition to chemotherapy, local therapies for primary and metastatic tumors should also be considered as important components of systemic therapy.

CN remains an important therapeutic modality for local therapy of primary tumors. In the era of IFN‐based systemic therapy, immediate CN followed by IFN improved survival compared to treatment with IFN alone.^8^, ^9^ Along with the widespread use of molecular targeted therapy with TKI, the role and sequence of CN seems to have changed.^10^, ^11^ The CARMENA study^12^ revealed that DCN following presurgical sunitinib, compared to sunitinib alone, was not inferior in overall survival, and was rather beneficial in disease control rate. The SURTIME study^13^ revealed that DCN following sunitinib, compared to immediate CN followed by sunitinib, was beneficial not only in overall survival but also in selecting out the patients with inherent resistance. The optimal sequence of CN and systemic therapy remains unclear in the era of IO therapy. However, because IO‐based combination therapies have demonstrated their superiority to sunitinib, not only immediate CN but also DCN should be considered for certain patients, in accordance with the CARMENA and SURTIME results.

In the present case, we offered first‐line chemotherapy with lenvatinib and pembrolizumab after comprehensive consideration of the patient. First, the primary tumor was so huge that we assumed immediate CN as quite an invasive therapeutic option. Second, since there were multiple metastases in both lungs, total metastasectomy might have led to excessive pulmonary function loss. A therapeutic strategy targeting CR was desirable. Among the IO‐based combination therapies, lenvatinib plus pembrolizumab and cabozantinib plus nivolumab combination have shown remarkable tumor objective response rates.^4^, ^5^ We expected the IO‐TKI combination to reduce the tumor volume remarkably, so that subsequent local therapies could be less invasive and result in CR. In addition, since cabozantinib can be used as a second‐line treatment in the Japanese public health insurance system, we could offer this TKI in case the first‐line lenvatinib plus pembrolizumab treatment failed. In the administration of lenvatinib, we set the starting dose at 14 mg to avoid the interruption due to the adverse events, with reference to the median dose intensity 13.9 mg within the overall population in CLEAR study.^5^ However, since whether the starting dose of TKIs should be reduced is still unclear, further accumulation of findings in the real world is essential. As a result, this combined treatment led to the complete disappearance of the metastatic lung tumors without metastasectomy. Moreover, the remarkable shrinkage of the primary tumor resulted in less‐invasive surgery using robot‐assisted laparoscopy and surgical CR. While prospective randomized clinical trials are necessary to confirm the role of DCN following IO‐based combination therapy, we believe that present case contributes to the accumulation of knowledge and helps clinicians for decision‐making in mRCC treatment.

## Author contributions

Hiroaki Sato: Conceptualization; data curation; formal analysis; investigation; methodology; project administration; resources; software; supervision; validation; visualization; writing – original draft; writing – review and editing. Tomokazu Sazuka: Conceptualization; data curation; formal analysis; investigation; methodology; project administration; supervision; writing – review and editing. Ayumi Fujimoto: Data curation; formal analysis; methodology; supervision; validation; writing – review and editing. Sakurako Kagitani: Investigation; methodology; supervision; visualization. Takayuki Arai: Conceptualization; data curation; formal analysis; project administration; supervision; validation. Yusuke Goto: Conceptualization; data curation; formal analysis; project administration; supervision; validation. Yusuke Imamura: Supervision; validation. Shinichi Sakamoto: Supervision; validation. Jun‐Ichiro Ikeda: Formal analysis; methodology; supervision; visualization. Tomohiko Ichikawa: Project administration; supervision; writing – review and editing.

## Conflict of interest

The authors have no conflicts of interest regarding to this case report.

## Approval of the research protocol by an Institutional Reviewer Board

This study is approved by our institutional ethic reviewer board and the approval number is 2554.

## Informed consent

An informed consent from the patient was obtained.

## Registry and the Registration No. of the study/trial

The registration number in a public trials' registry is not applicable for this study.
